# Transcranial direct current stimulation on social communication among children and adolescents with autism spectrum disorder: a systematic review and meta-analysis

**DOI:** 10.1186/s11689-026-09672-6

**Published:** 2026-01-20

**Authors:** Maryam Alabbad, Shibili Nuhmani, Raafat Ahmed, Shahid Bashir, Muhammad Ajmal Khan, Turki Abualait

**Affiliations:** 1 College of Applied Medical Sciences, Imam Abdulrahman Bin Faisal University, P.O.Box 2435, Dammam, 31451 Saudi Arabia; 2 Neuroscience Center, King Fahad Specialist Hospital, Dammam, Saudi Arabia; 3 Deanship of Library Affairs, Imam Abdulrahman Bin Faisal University, Dammam, Saudi Arabia

**Keywords:** TDCS, ASD, Social Cognition, Emotion recognition, Theory of mind, Meta-analysis

## Abstract

**Background:**

Transcranial direct current stimulation (tDCS) has gained attention as a potential intervention to improve social cognition in children with autism spectrum disorder (ASD). However, its effects across social domains and the influence of stimulation parameters remain unclear. This systematic review and meta-analysis evaluate the effectiveness of tDCS in enhancing social functioning in children with ASD, focusing on emotion recognition, theory of mind (ToM), and social responsiveness.

**Methods:**

A comprehensive search identified randomized controlled trials (RCTs) investigating tDCS effects on social cognition in children with ASD. Studies were assessed for effect sizes and statistical significance. A meta-analysis pooled results, and moderators of tDCS effectiveness were examined.

**Results:**

Screening 14 studies revealed that anodal tDCS applied to the left dorsolateral prefrontal cortex (DLPFC) produced the most consistent improvements in emotion recognition, ToM, and social responsiveness. However, results varied, with some studies reporting improvements, while others showed no substantial effects. Dual-stimulation or cathodal stimulation yielded mixed outcomes. Evidence was limited by small sample sizes, risk of bias, and variability in stimulation parameters.

**Conclusions:**

Anodal tDCS over the left DLPFC shows promise for improving social cognition in ASD. Larger controlled trials are needed to determine the effectiveness of combining tDCS with social skills training.

## Highlights

• The left DLPFC is the top targeted brain region for enhancing social cognition in children with ASD.

• Emotional recognition, theory of mind, and social responsiveness scale are the most tasks utilized.

• Dual stimulation protocols, combining anodal and cathodal tDCS stands out as the most promising stimulation intervention for social cognition enhancements.

• Further research with large-scale randomized trials and standardized protocols is needed to optimize tDCS efficacy for children with ASD.

## Introduction

Autism Spectrum Disorder (ASD) is a neurodevelopmental disorder characterized by deficiencies in social interface, accompanied by limited and recurring activities. The Centers for Disease Control and Prevention (CDC) indicates that the incidence of ASD is around 1–2%, with a significant rise in recent decades. The disease is now shown to be fourfold more common in males than in females [1]. The increase in prevalence may not only indicate a genuine rise in instances but might also result from improved diagnostic criteria or advanced assessment methods [2, 3]. Deficiencies in social perception and social abilities are essential characteristics of ASD. Social cognition encompasses the intellectual processes needed for comprehending and maneuvering through social contexts [4], while social skills pertain to the practical use of these cognitive processes to participate proficiently in social interactions [5].

Social issues challenged by persons with ASD provide enduring difficulties of establishing and sustaining meaningful relationships [6], obtaining satisfying work, and attaining independent living [7]. The challenges are most pronounced in interactions requiring emotional processing [8] and in the ability to recognize and articulate emotions, which are closely linked to ASD-related impairments in empathy [9, 10]. This impairment is also associated with difficulties in theory of mind (ToM), which is the capability to comprehend others' viewpoints and deduce their mental states. Prior cognitive neuroscience research indicates that impairments in ToM, executive processes, and emotion control contribute to the fundamental social and interaction problems correlated with ASD [11–14]. ToM denotes the capacity to ascribe intellectual states, such as emotions or goals, to oneself and others [15]. ToM hypothesis suggests that persons with ASD have considerable deficits in social perception, especially in cognitive empathy and the comprehension of others' cognitive states [16]. Due to its pivotal importance in social competencies, several recent research has focused on the ToM in ASD [17–19].

ASD causes a substantial burden on both the individual's quality of life and healthcare expenditures [20]. This highlights the continual need to investigate and evaluate diverse treatment alternatives for ASD. Psychotherapy is the favored treatment method, especially for tackling social-communication difficulties. Nevertheless, even the most proven early behavioral therapies or social skills training programs often provide only small to moderate enhancements in social reciprocity and responsiveness [21–23]. Moreover, no pharmacological treatment explicitly addressing social communication problems in ASD has shown efficacy yet. There is an urgent necessity for additional effective and competent interventions for ASD.

At present, no pharmaceutical interventions have shown efficacy in alleviating the fundamental symptoms of compromised social interface and interaction in ASD. Behavioral therapies in early life, especially during infancy and toddlerhood, have shown a modest impact on social mutuality in ASD [23]. Furthermore, autism-targeted social abilities educating has shown minor to moderate enhancements in social responsiveness among older children and adolescents with high-functioning ASD [21, 22]. Significant diversity in individual responses has been seen in the majority of psychotherapy intervention trials, with overall effect sizes generally ranging from moderate to medium. Therefore, there is a substantial need for novel therapy strategies that address the neurological underpinnings underlying the illness. Noninvasive transcranial stimulation presents a viable method to enhance therapy designed to improve social perception and social abilities [24].

Transcranial direct current stimulation (tDCS) is a non-invasive neuromodulation technique that delivers low-amplitude direct current via scalp electrodes to modulate cortical excitability [25]. The effects depend on electrode polarity, montage, and stimulation parameters. Anodal tDCS typically increases cortical excitability under the anode, while cathodal tDCS generally decreases excitability under the cathode [25, 26]. This polarity-dependent modulation is supported by both conventional and high-definition (HD) tDCS studies, with HD-tDCS offering greater spatial precision in targeting cortical regions [26–30]. Dual stimulation refers to montages where both anodal and cathodal electrodes are placed over distinct cortical targets, allowing for simultaneous up- and down-regulation of excitability in different regions, though most clinical and research protocols use single-target stimulation [25, 26].

The primary underlying mechanism of tDCS effects involves subthreshold polarization of neuronal membranes, which can alter synaptic efficacy and plasticity [25, 26]. tDCS does not directly elicit action potentials but modulates neuronal membrane potentials, thereby influencing the likelihood of neuronal firing [25, 26]. Anodal tDCS stimulation depolarizes neurons, facilitating excitatory neurotransmission, while cathodal stimulation hyperpolarizes neurons, reducing excitability [25]. These effects are mediated by changes in glutamatergic and GABAergic transmission and can induce neuroplastic changes via NMDA receptor-dependent mechanisms [26, 31, 32]. The modulation of alpha and beta EEG power following tDCS further supports its impact on cortical oscillatory activity [33].

Regarding cephalic reference electrode placement, conventional tDCS typically uses large sponge electrodes with the active electrode over the target region and the reference electrode over a cephalic site (e.g., contralateral supraorbital area) [25]. This results in a diffuse electric field affecting broad cortical areas [27, 29, 33]. In contrast, for extracephalic reference placement, the reference electrode is placed on a non-cephalic site (e.g., shoulder or arm), which can reduce confounding effects on non-targeted brain regions but may alter current flow and safety profile [33]. This approach is less common and requires careful consideration of current density and impedance [33].

High-definition tDCS (HD-tDCS) employs arrays of smaller electrodes (e.g., 4 × 1 ring configuration: one central electrode surrounded by four return electrodes) to achieve more focal stimulation [31]. Physiological, computational, and modeling studies have yielded converging evidence regarding the focality, current distribution, and network-level effects of tDCS, establishing the foundation for high-definition stimulation methodologies [34–37]. Computational modeling and empirical studies confirm that HD-tDCS produces a more localized and directionally precise electric field, with the region of influence and focality adjustable by electrode configuration and ring diameter [27, 28]. HD-tDCS protocols typically use 1–2 mA for 10–20 min, with electrode placement guided by anatomical or functional imaging to optimize targeting [27–30, 38]. tDCS effects are polarity-dependent, mediated by subthreshold modulation of neuronal membrane potentials and synaptic plasticity, and are highly sensitive to electrode montage. HD-tDCS offers superior focality and spatial precision compared to conventional approaches, as demonstrated by both computational and neurophysiological evidence [27–30, 38].

Genetic syndromes such as ASD can alter the effects and outcomes of cortical tDCS through several neurobiological and network-level mechanisms unique to these populations [39, 40]. In ASD, there are well-documented alterations in cortical excitability, excitation/inhibition (E/I) balance, and functional connectivity, all of which can influence both the neurophysiological response to tDCS and its clinical efficacy [40]. First, individuals with ASD often exhibit disrupted E/I balance, particularly in prefrontal and midline cortical circuits. tDCS protocols—both anodal and cathodal—have been shown to modulate this balance in ASD, with evidence that cathodal prefrontal tDCS can reduce elevated theta-band E/I ratios and improve information processing efficiency and social functioning, suggesting that the baseline neurophysiological state in ASD may render these circuits more responsive to tDCS-induced modulation than in neurotypical populations [41]. Second, the presence of sensory processing abnormalities in ASD can moderate tDCS outcomes. For example, high-definition tDCS (HD-tDCS) targeting social impairment in ASD demonstrates greater efficacy in children with typical or hypo-tactile sensory profiles, while those with hyper-tactile sensitivity show more limited benefit. This indicates that underlying sensory integration phenotypes, which are often genetically determined in ASD, can influence both the magnitude and the domains of tDCS response [42]. Third, tDCS in ASD has been shown to induce changes in brain network dynamics that differ from those observed in neurotypical controls. EEG and network analyses reveal that tDCS can increase both static and dynamic functional connectivity in ASD, partially normalizing the atypical network patterns characteristic of the disorder [40, 43]. These network-level effects may underlie observed improvements in social cognition, theory of mind, and emotion regulation following dorsolateral prefrontal cortex (DLPFC)-targeted tDCS [44]. Fourth, tDCS induces measurable changes in brain metabolites, and these effects are dependent on the polarity of stimulation [25]. Anodal tDCS, which increases cortical excitability, has been shown to elevate N-acetylaspartate (NAA)/creatine (Cr) and myoinositol (mI)/Cr ratios, while decreasing choline (Cho)/Cr concentrations in targeted regions such as the left dorsolateral prefrontal cortex (DLPFC) and locus coeruleus in individuals with autism spectrum disorder (ASD) [39]. These metabolite shifts are interpreted as markers of enhanced neuronal integrity (NAA), glial activity (mI), and altered membrane turnover (Cho), reflecting underlying changes in synaptic and cellular function. In healthy adults, cathodal stimulation may also trend toward increased NAA/tCr in the motor cortex, suggesting that both anodal and cathodal tDCS can modulate neurometabolic profiles, though the direction and magnitude of change may differ by polarity and brain region [45]. In contrast, ASD is characterized by synaptic dysfunction, often involving aberrant NMDA receptor-mediated plasticity and mGluR signaling, which can disrupt normal metabolite profiles and energy regulation in cortical circuits. The observed tDCS-induced metabolite changes in ASD may therefore reflect a normalization or compensation of these underlying synaptic and glial abnormalities, potentially contributing to clinical improvements in social functioning [39]. However, because ASD-associated genetic variants can alter the baseline state of synaptic and metabolic pathways, the response to tDCS, including the direction and magnitude of metabolite changes, may differ from that seen in neurotypical individuals or other neuropsychiatric populations [45]. This highlights the importance of considering ASD-specific neurobiology when interpreting tDCS effects and designing therapeutic protocols. Finally, clinical outcomes in ASD following tDCS are variable and appear to depend on stimulation parameters, the presence or absence of concurrent cognitive training, and the specific cognitive or behavioral domains targeted. For example, repeated anodal tDCS to the left DLPFC can improve non-verbal intelligence and core ASD symptoms, but the effect size and durability may differ from those seen in neurotypical populations, likely reflecting the unique neurodevelopmental context of ASD [44, 46]. Overall, the neurobiological and network alterations inherent to ASD—including E/I imbalance, sensory processing phenotypes, and atypical functional connectivity—modulate both the physiological and clinical effects of tDCS. These factors must be considered when designing and interpreting tDCS interventions in ASD and other genetic syndromes [39–44, 46]. Clinical and experimental studies examining tDCS in autism spectrum disorder have demonstrated inconsistent effects on social cognition, emotion regulation, and functional connectivity, indicative of variability in stimulation protocols, targets, and outcome measures [47–50].

In ASD, the social-cognitive network involves key regions including the superior temporal sulcus (STS) and the temporoparietal junction (TPJ). The TPJ is a fundamental area implicated in social cognition, theory of mind, and facial emotion processing. It has been consistently examined through non-invasive brain stimulation techniques, such as conventional and high-definition transcranial direct current stimulation (tDCS), to investigate the causal mechanisms underlying these functions [51–54]. Converging evidence have shown that the TPJ consistently exhibits reduced neural activation and diminished functional connectivity [55, 56]. Moreover, the DLPFC as a key region in the neural circuitry underlying emotion recognition and social cognition, acting as a hub to control limbic responses and modulate affective processing [57, 58], exerts top-down control over emotional processing, modulating the valence attribution of emotional experiences and influencing the integration of higher-order semantic information during emotional perception [57, 58]. Evidence from lesion and neuroimaging studies demonstrate that the DLPFC is essential for accurately identifying others' emotions and for efficient processing of social cues; damage to this region leads to deficits in both the accuracy and speed of emotion recognition tasks, as well as altered electrophysiological responses during social perception [59]. Functional MRI studies further show that the DLPFC is activated during the detection of incongruency in social interactions, such as when evaluating whether an individual's actions align with social expectations, highlighting its role in context-dependent social judgment [60]. Collectively, these findings establish the DLPFC as a central hub for the cognitive control of emotion, context-sensitive social evaluation, and the integration of complex social information, all of which are foundational for emotion recognition and social cognition [57–60].

Yet, no systematic review and meta-analysis have particularly investigated the effect of tDCS on social interactions in children with ASD. This study is to analyze the current research to evaluate the efficacy of tDCS as a possible intervention for enhancing social interactions in this demographic. We aim to integrate data via a systematic review and meta-analysis, elucidating the impacts of tDCS on social behaviors in children with ASD, while emphasizing the strengths and limitations of existing research.

## Methods

This review was performed in compliance with the Cochrane Handbook for Systematic Reviews of Interventions and the Preferred Reporting Items for Systematic Reviews and Meta-Analyses (PRISMA 2020 Checklist). A pre-established, documented procedure for this systematic review was submitted to the PROSPERO platform (www.crd.york.ac.uk/PROSPERO/) under the identifier CRD42024604148.

### Selection criteria

All records retrieved from the database searches were uploaded into the citation management software Endnote X9, where duplicate records were removed automatically, and additional duplicates were eliminated manually. Table 1 outlines the criteria for inclusion and exclusion depending on the PICO Model. This review considered both randomized controlled trials (RCTs) and non-RCTs published in peer reviewed journals in English language. Studies were included if they examined the impacts of tDCS on Children and adolescents diagnosed with ASD according to standardized criteria, such as the DSM-5. Only studies addressing pure ASD without any additional diagnoses were considered. These studies utilized tDCS as the primary intervention, targeting improvements in social interaction. Relevant outcomes included aspects of social interaction (e.g., social responsiveness, communication skills), assessed using standardized evaluation tools. The exclusion criteria, also summarized in Table 1, included case reports, reviews, meeting abstracts, editorials, proceedings articles, letters to the editor, book chapters, conference abstracts, and pilot studies. Additionally, studies involving individuals with ASD who had comorbid conditions (e.g., attention-deficit/hyperactivity disorder (ADHD)) were excluded, as well as studies focusing on gene interactions and heritability in ASD.

**Table 1:** Inclusion and Exclusion Criteria for ASD Intervention Studies

Category	Inclusion criteria	Exclusion criteria
Population	Children and adolescents aged less than 18 years; diagnosed with ASD	ASD with comorbidities such as (attention deficit hyperactivity disorder “ADHD is multifactorial and highly heritable; multiple genes and non-inherited factors contribute”)
Intervention/Assessment measures	tDCS	–––––
Comparator	Any	–––––
Outcome measures/	Social interaction (e.g., social responsiveness, communication skills)	Genes
Study design	RCTs, non-RCTs (clinical trials, cohort, prospective, and experimental studies)	Case reports, reviews, meeting abstracts, editorials, proceedings articles, letters to the editor, book chapters, conference abstracts without complete reports, note, and pilot research

### Search strategy

The four electronic databases—Medline, Web of Science, Scopus, and Embase—were searched by two authors (MA and SN) for studies published in English from inception to November 2024. An exhaustive search was conducted using Medical Subject Headings (MeSH) combined with text keywords and Boolean operators. A systematic search was conducted across multiple databases, including MEDLINE, Web of Science, Scopus, and Embase, using relevant keywords such as 'transcranial Direct Current Stimulation', 'autism', and 'social interaction'. The search strategies were tailored for each database to ensure comprehensive coverage of the literature. The full search queries for each database can be found in (Table 2).

**Table 2:** Search Queries Used in Databases

Database	Search Query
MEDLINE	TS = ("transcranial Direct Current Stimulation" OR tSCDS) AND TS = (autism OR Autistic OR "Pervasive Developmental Disorder" OR PDD OR ASD OR "Asperger Syndrome" OR "Kanner’s Syndrome" OR "Neurodevelopmental Disorder") AND TS = (social*)
Web of Science	TS = ("transcranial Direct Current Stimulation" OR tSCDS) AND TS = (autism OR Autistic OR "Pervasive Developmental Disorder" OR PDD OR ASD OR "Asperger Syndrome" OR "Kanner’s Syndrome" OR "Neurodevelopmental Disorder") AND TS = (social*)
Scopus	TITLE-ABS-KEY("transcranial Direct Current Stimulation" OR tdcs) AND TITLE-ABS-KEY(autism OR autistic OR "Pervasive Developmental Disorder" OR pdd OR asd OR "Asperger Syndrome" OR "Kanner’s Syndrome" OR "Neurodevelopmental Disorder") AND TITLE-ABS-KEY(social*)
Embase	(("transcranial Direct Current Stimulation" OR tdcs)) AND ((autism OR autistic OR "Pervasive Developmental Disorder" OR pdd OR asd OR "Asperger Syndrome" OR "Kanner’s Syndrome" OR "Neurodevelopmental Disorder")) AND (social*)

The studies were systematically arranged in the references list to aid the reviewers in performing study selection. Initially, studies were evaluated by title and abstract to determine compliance with inclusion criteria, along with reference lists from pertinent systematic reviews were also searched for additional articles.

### Data extraction

Data were independently extracted by two reviewers (MA and SN) using a standardized data extraction form developed in Microsoft Excel. The extracted information included study design, participant demographics, intervention details, outcome measures, and results. To ensure accuracy, the first author compiled the relevant data, while the second author verified it. Titles and abstracts were initially screened, and full-text articles were re-evaluated. If any article lacked sufficient information, the authors contacted the original study authors for clarification. A third reviewer (TA) helped resolve any discrepancies, ensuring consensus in the inclusion and exclusion criteria throughout the process.

### Quality assessment

Two authors (MA and SN) carried out an independent reliability assessment to evaluate potential bias in the included studies. To assess bias in the RCTs, the Cochrane Risk of Bias 2 (RoB2) was utilized. This tool evaluates five critical areas of possible bias: randomization techniques, variations from the planned intervention, missing data, outcome measurement, and reported result selection. The ROBINS-I tool was used to evaluate nonrandomized studies. This tool evaluates the risk of bias owing to confounding, selection bias, intervention categorization, variations from the planned intervention, missing data, outcome measurement, and result selection based on reported results. Two reviewers (MA and SN) classified all eligible studies according to their risk of bias as high, low, or 'some concern,' and any conflicts were addressed via discussion with a third reviewer (TA).

### Statistical analysis

A meta-analysis was conducted for studies with similar objectives, interventions, and outcomes, while a narrative synthesis was used for others. In the meta-analysis, the standardized mean difference (Hedges g) and standard error were calculated for each study. All meta-analyses were conducted with Cochrane's RevMan 5.3. Continuous data were shown as mean differences (MDs) with 95% confidence intervals (CIs), and dichotomous data as relative risk with 95% CIs. Statistical heterogeneity was measured with the Cochrane Q test (*P* < 0.1 for significance) and quantified using the I^2^ statistic. An I^2^ value under 50% indicated no significant heterogeneity, and the fixed-effect model was used. When high heterogeneity was found, random-effects models or subgroup analyses were conducted to identify potential sources of variation or perform sensitivity analyses. Sensitivity analyses aimed to improve findings by removing studies with high bias risk, missing data, or outliers. Publication bias was assessed with funnel plots and Egger's test for asymmetry. Subgroup analyses investigated factors such as control groups, population characteristics, delivery mode, treatment duration, and risk of bias to examine sources of heterogeneity and treatment effect variations.

We applied the Grades of Recommendation, Assessment, Development, and Evaluation (GRADE) system [61] to assess the quality of the evidence and the strength of the recommendations, utilizing GRADEpro software [62] to generate the table. The quality of the evidence was rated on a four-point scale: ‘high’, ‘moderate’, ‘low’, and ‘very low’.

## Results

### Study selection

**Fig. 1:**
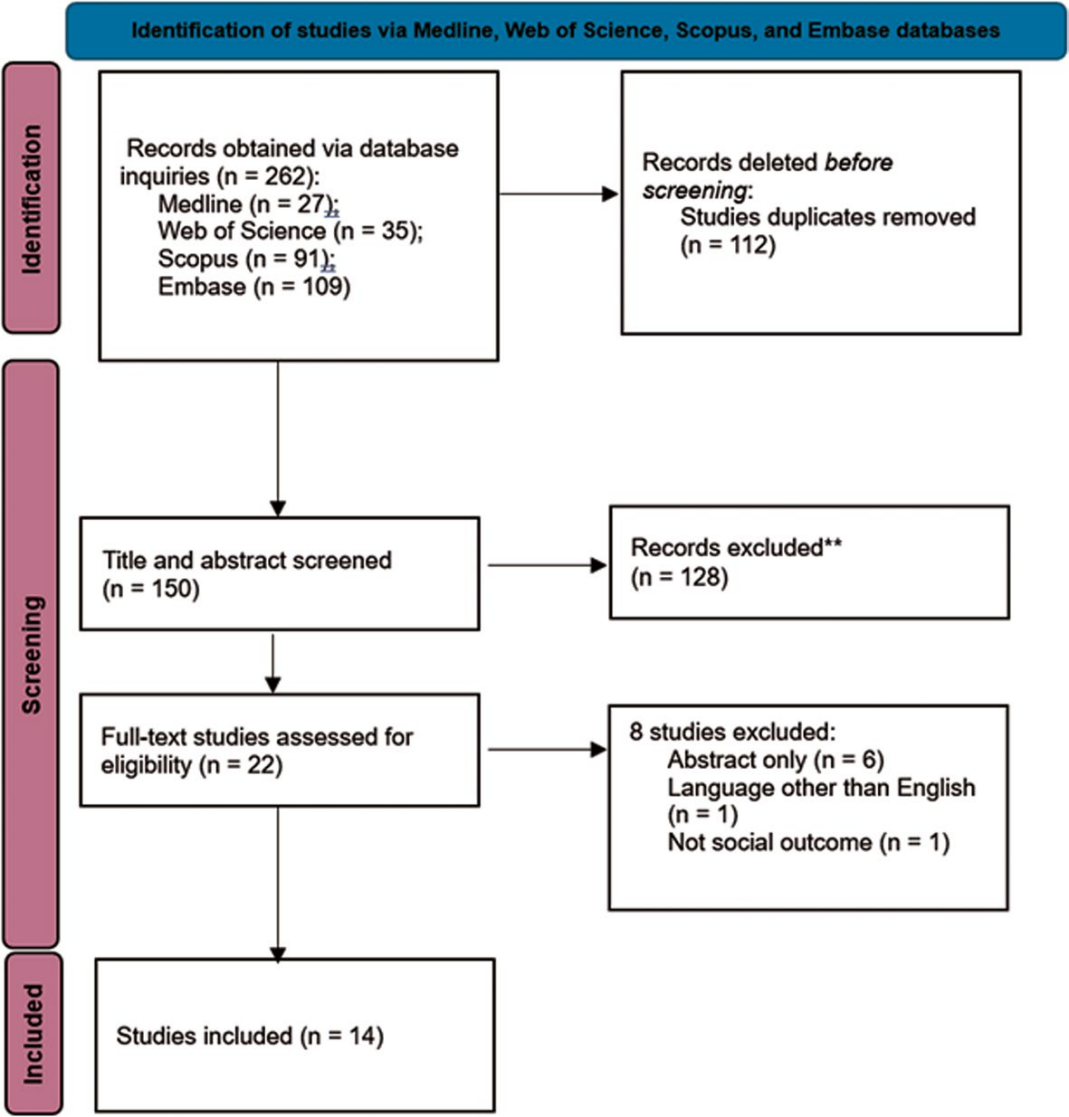
PRISMA Flow Diagram. The study choosing process in the systematic review is shown in the PRISMA flow diagram. It lists at every level the recognized, included, and deleted records. Two hundred sixty-two papers first came from electronic databases. Twenty-two full-text papers were screened for eligibility after title and abstract based removal of duplicates. In the end, fourteen papers satisfied the inclusion criteria and were included into the review

This review presents the results of electronic searches conducted up to November 6, 2024. A total of 262 study titles were initially screened, and after removing duplicates and conducting title and abstract screening, 22 full-text articles were evaluated for eligibility. Of these, eight were omitted for the following reasons: six were only available as abstracts, one was not published in English, and one did not examine social interaction as an outcome. A full-text review of the remaining 14 studies was conducted independently by two authors, and all 14 studies met the inclusion criteria, being included in the final review. The criteria for study selection and the rationale for exclusion are outlined (Fig. 1).

### Study characteristics

Fourteen studies were included in this review that examined the impacts of tDCS on social functioning in individuals diagnosed with ASD [40, 41, 44, 63–72] (Table 3). The studies were carried out in Thailand [63], Hong Kong [41], Cuba [64], China [40, 65–67, 71, 72], Iran [13, 14, 44, 68], and Austria [69, 70]. The included studies employed different designs, including randomized controlled trials (RCT), sham-controlled trials, controlled trials, and quasi-experimental designs. The mean age of participants across the studies varied from 6.5 to 17.1 years. Eight studies included both male and female participants [13, 14, 40, 41, 63, 65–67, 71, 72], Two studies only included males [44, 68], and three studies did not specify participant sex [64, 69, 70]. Sample sizes in the studies varied from 10 to 60 participants. The left DLPFC was the most common target for stimulation [40, 41, 44, 63–67, 71, 72]. Anodal stimulation was used most often [40, 44, 63, 64, 67, 68, 71, 72], while some studies used cathodal stimulation [41, 64, 66] or a combination of both [13, 14, 44, 65]. The intensity of stimulation ranged from 1 to 2 mA, with a typical duration of 20 min per session. The number of tDCS sessions in the studies ranged from a single session to 20 sessions, which were delivered over a period of days or weeks. Participants in some studies continued to receive their conventional treatment for ASD, such as speech therapy, occupational therapy, and medication, while participating [63, 65, 66]. Some studies paired the tDCS intervention with cognitive training [41, 65, 66, 69–71]. Most studies reported that tDCS was well-tolerated, with the most reported side effects being mild and transient [41, 64]. Itching, trouble concentrating, and headaches were the most common side effects [41, 66]. Study-level details are summarized in (Table 3).

**Table 3:** Characteristics of included studies (*n* = 14)

*Author (year)*	Study design	Experimental group		Control group		tDCS methodology						Outcomes Measures	Results
		**N°**	**Mean age** **(range)**	**N°**	**Mean age** **(range)**	**Stimulation**	**Intensity**	**Treatment duration**	**N° sessions**	**Anodal location**	**Cathodal location**		
Kang et al. [67]	Controlled trial design	13	Mean 6.5 y (SD = 1.7)	13	Mean 6.3 y (SD = 1.7)	Mixed	1 mA	20 min	10 every other day	DLPFC	Right supraorbital	Normalized maximum entropy ratio (NMER), maximum entropy ratio (MER) algorithm	NMER value significantly increased
Nazari et al. [68]	Quasi-experimental pretest–posttest design with intervention and control groups	10	Mean 9 y (SD = 2.36), Range 6–17 y	11	9.81 y (SD = 2.4), Range 6–17 y	Anodal	2 mA	15 min	10 sessions, 72-h interval between sessions	Left DLPFC	Right DLPFC	Emotion recognition task and the ATEC	Significant improvement in emotion recognition scores, ATEC scores did not show statistically significant changes
Zemestani et al. [44]	Randomized, double-blind, sham-controlled, parallel-group	17	Mean 8.0 y (SD = 1.83), Range 7–12 y	15	Mean 8.2 y (SD = 2.48) Range 7–12 y	Mixed	1.5 mA	15 min	10, 2 times per week	Left DLPFC (F3)	Right DLPFC (F4)	GARS-2, ToM Test, Emotion Regulation Checklist (ERC), Conners’ Parent Rating Scale-Revised (CPRS-RS)	Positive (communication domain, ToM, and in emotion regulation strategies)
Prillinger et al. [70]	Randomized, double-blind, and sham-controlled clinical trial	11	Mean 14.1 y (SD = 1.9)	11	Mean 14.1 y (SD = 1.9)	Anodal	1 mA	20 min	10	F3	Right supraorbital	Movie for the Assessment of Social Cognition (MASC), emotion recognition task (ERT), and eye-tracking data	Both the active and sham tDCS groups showed progresses in emotion recognition accuracy, with no significant differences between them
Zhou et al. [40]	Controlled trial	18	Mean 6.5 y (SD = 1.4)	18	Mean 6.7 y (SD = 1.3)	Anodal	1 mA	20 min	single session	Left DLPFC (FC3)	Right eyebrow	Brain network alterations using network-based methodologies	Significantly altered functional connectivity and rapid changes in the brain's resting-state organization
Salehinejad et al. [13, 14]	Randomized, sham-controlled, cross-over	16	Mean 10.7 y (SD = 1.9)	16	Mean 10.7 y (SD = 1.9)	Mixed	1.5 mA	20 min	3 with a 72-h interval between sessions	Right temporoparietal cortex (r-TPJ)	Ventromedial prefrontal cortex (vmPFC)	Theory of Mind (ToM) task	No significant improvement in ToM performance compared to sham
Wang et al. [72]	RCT	30	4–12 y	15	4–12 y	High-definition tDCS (HD-tDCS)	1.5 mA	20 min	14 (daily over 3 weeks)	Cz central anode, F3 central anode	Left DLPFC, F3	Social Responsiveness Scale-Chinese Version (SRS-Chinese Version) and parental reports on sleep quality	Significant improvements in SRS domains, including total score, autism behavior, and social communication
Chan et al. [41]	2-armed, parallel RCT	30	Mean 16.79 years (SD = 2.32), Range 14–21 y	30		Cathodal	1.5 mA	20 min	10	Left DLPFC, Fp2	Left DLPFC, F3	Social Responsiveness Scale-2nd Edition (SRS-2), information processing efficiency (IPE), and electrophysiological measures of excitation/inhibition (E/I) balance	Significant improvements in social functioning, as indicated by diminished SRS-2 total and RRB domain scores, and enhanced IPE
Amatachaya et al. [63]	Randomized, double-blind crossover, sham-controlled trial	20	6.4 y (5–8 y)	20	6.4 y (5–8 y)	Anodal	1 mA	20 min	Over 5 consecutive days, with a four-week washout between the active and sham phases	Left DLPFC, F3	On the shoulder contralateral to the anode	Peak alpha frequency (PAF) and autism-related problem domains	Increased PAF at F3 showed no effect on the ATEC total score, but correlated with improvements in social interaction on the ATEC social scale
Prillinger et al. [69]	Randomized, double-blind, and sham-controlled clinical trial	22	Mean 14.1 y (SD = 1.9)	22	Mean 14.1 y (SD = 1.9)	Anodal	2 mA	20 min	10 sessions over two weeks	Right F3	Right supraorbital	ERT	Significant improvements in emotion recognition accuracy, particularly for happy and fearful facial expressions
Han et al. [65]	2-armed, parallel RCT	21	Mean 17 y; range 14–21 y	20	Mean 17 y; range 14–21 y	Cathodal	1.5 mA	20 min	Over two consecutive weeks	Right supraorbital (Fp2)	Left DLPFC (F3)	SRS-2, the Wisconsin Card Sorting Test (WCST), and the CANTAB® 5-choice Reaction Time (RTI) task	Significant improvement in the IPE score, with no between-group differences in social functioning or CF
Gómez et al. [64]	Open trial design without the use of placebo stimulation	24	mean 12.2 y	0	12.2	Cathodal	1 mA	20 min	20 sessions (5 ×/week × 4 weeks)	Left DLPFC	Left DLPFC	Autism Diagnostic Interview-Revised (ADI-R), the Autism Behavioral Checklist (ABC), the Autism Treatment Evaluation Checklist (ATEC), and the Global Clinical Impression Scale (GCIS)	Significant decreases in total scores for the ADI-R, ABC, and ATEC scales
Sun et al. [71]	Single-blind, sham-controlled experiment	19	Mean 8.0 y (SD = 1.9), Range 4–12 y	18	Mean 7.6 y (SD = 2.1), Range 4–12 y	Anodal	1.5 mA	20 min	4 weeks, three times a week	Left DLPFC F3	Right supraorbital	MMN amplitude and latency, and ABC scale scores	Increased MMN amplitude for tone-deviant stimuli, correlated with improved ABC scale scores
Han et al. [66]	Triple-arm, double-blind, randomized clinical trial	34	Mean 17.05 y (SD = 2.48) Range 14–21	63	Mean 16.71 y (SD = 2.04) Range 14–21	Cathodal	1.5 mA	20 min	10	Fp2	F3	Social Responsiveness Scale-2nd edition (SRS-2) total, social communication, and RRB T-scores	Significant group*time interactions for SRS-2

*tDCS* Transcranial Direct Current Stimulation, *SD* Standard Deviation, *y* Years, *mA* Milliamps, *DLPFC* Dorsolateral Prefrontal Cortex, *NMER* Normalized Maximum Entropy Ratio, *MER* Maximum Entropy Ratio, *ATEC* Autism Treatment Evaluation Checklist, *GARS-2* Gilliam Autism Rating Scale-2, *ToM* Theory of Mind, *ERC* Emotion Regulation Checklist, *CPRS-RS* Conners' Parent Rating Scale-Revised, *MASC* Movie for the Assessment of Social Cognition, *ERT* Emotion Recognition Task, *SRS-2* Social Responsiveness Scale-2nd Edition, *E/I* Excitation/Inhibition, *PAF* Peak Alpha Frequency, *RTI* Reaction Time Task, *WCST* Wisconsin Card Sorting Test, *CANTAB* Cambridge Neuropsychological Test Automated Battery, *ADI-R* Autism Diagnostic Interview-Revised, *ABC* Autism Behavioral Checklist, *GCIS* Global Clinical Impression Scale, *MMN* Mismatch Negativity

### Risk of bias among included studies

**Fig. 2:**
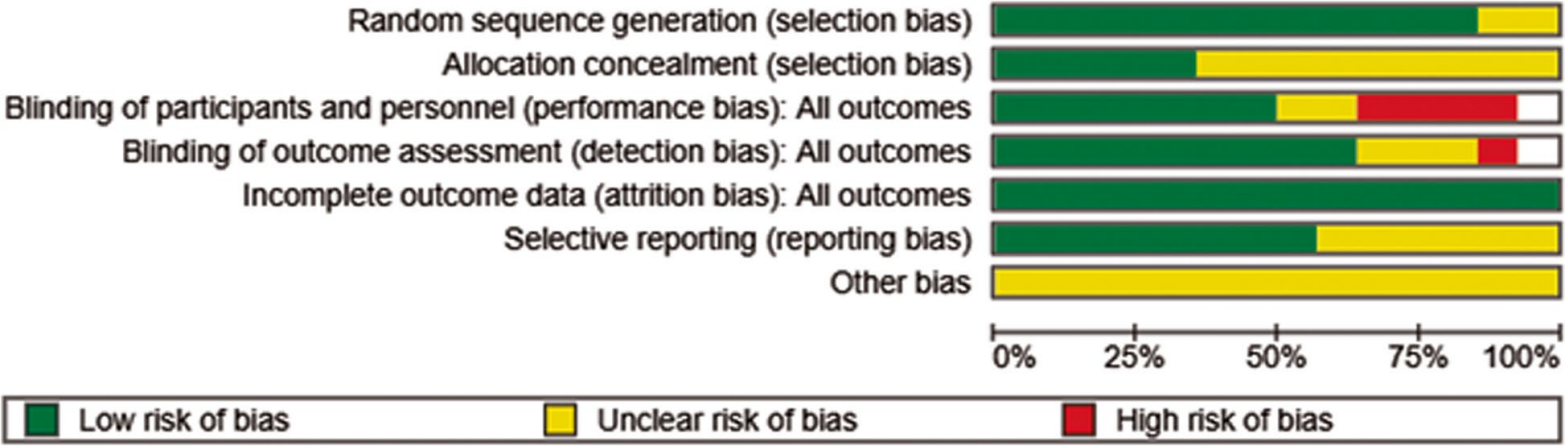
Graph of Quality Assessment—Risk of Bias. The risk of bias graph shows the proportion of studies with low, high, or unclear risk of bias across several spheres, so providing the general quality assessment of the included studies

**Fig. 3:**
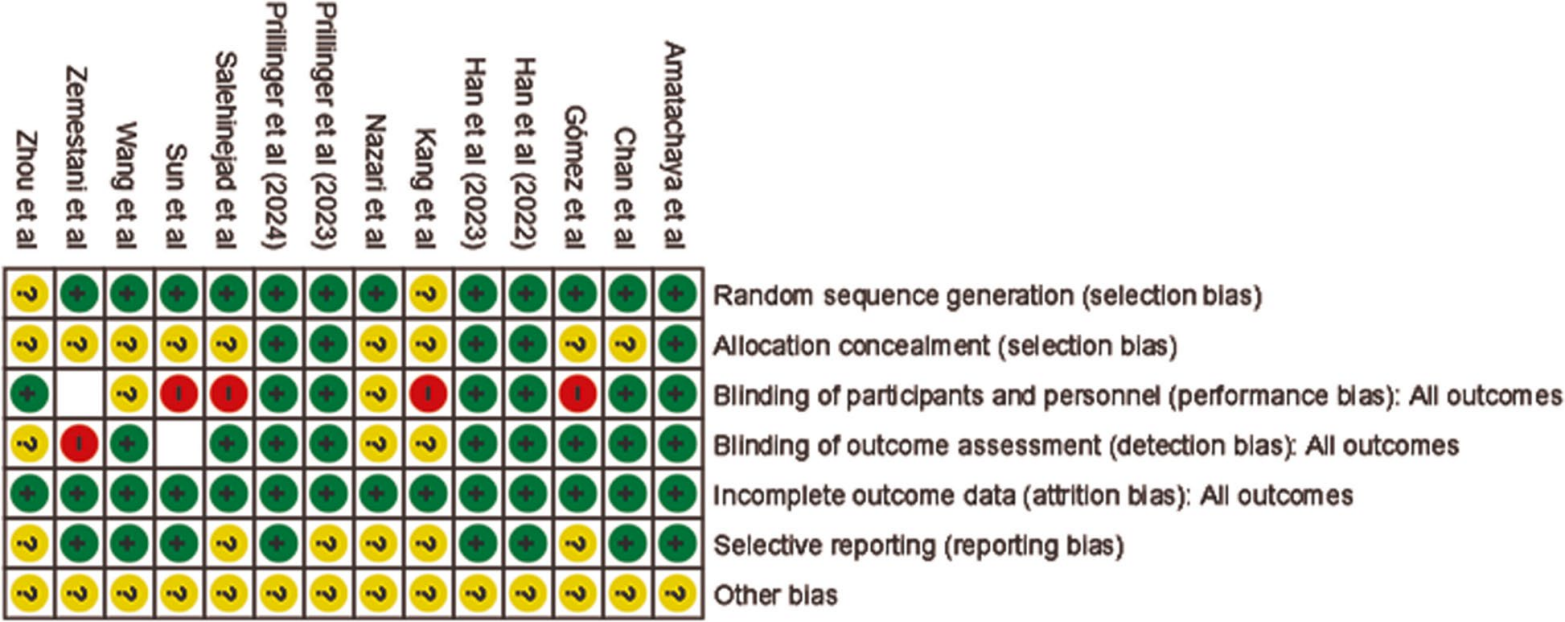
Risk of Bias Summary—Quality Assessment. By means of a comprehensive risk of bias assessment for every included study, this summary chart highlights possible methodological issues in individual assessments

The risk of bias was found to be moderate to high for most of the included studies. The primary source of bias stemmed from difficulties in blinding both participants and personnel, mainly due to the perceptible sensations associated with tDCS. This issue was widespread across the studies, as participants could often identify their treatment allocation, making it challenging to achieve a low risk of bias. The few studies that achieved a low risk of bias were those that used active controls to effectively blind participants to their group assignment [13, 14, 41, 63, 64, 66, 68–70, 72]. Although deviations from the planned interventions were rare, missing data resulting from participant dropout was a common issue. Information on attrition was not always provided clearly, and dropout rates ranged from 0 to 50% across studies. Most of the controlled trials showed higher dropout rates in the intervention groups compared to the control groups, suggesting that participants receiving active tDCS may have experienced more side effects or found the intervention more demanding [13, 14]. However, one study found the control group had a dropout rate twice that of the intervention group, possibly due to participant dissatisfaction with being assigned to the control group. This problem was reduced in other studies using waitlists or active control groups. The overall risk of bias for all 14 included studies is shown visually in (Fig. 2). A domain-wise summary of risk of bias is shown in Fig. 3.

### Findings on the effectiveness of tDCS in enhancing social interactions

This systematic review synthesizes the results from 14 studies examining the effects of tDCS on social functioning in children with ASD. The review focuses on the strength and statistical significance of these effects, as measured by effect sizes and *p*-values, across social domains such as social interactions, emotion recognition, ToM, and communication.

#### Studies examining social interactions

In terms of social functioning, tDCS interventions yielded varying effects across studies. Chan et al. [41] demonstrated significant improvements in social functioning following tDCS, with a moderate effect size (η^2^ = 0.105) and a notable group-by-time interaction (F(1, 58) = 6.79, *p* = 0.012). Improvements were primarily linked to reductions in restricted and repetitive behaviors (RRBs), rather than enhanced social communication. Similarly, Han et al. [65] observed notable improvements in social functioning after 10 sessions of prefrontal tDCS, with a small to moderate effect size on the Social Responsiveness Scale (SRS-2) total score (F(1, 39) = 4.75, *p* = 0.035), indicating positive effects on both social communication and RRBs. However, Prillinger et al. [70] reported no significant advancements in emotion recognition following 10 sessions of tDCS, as both active and sham groups exhibited similar gains. In contrast, Prillinger et al. [69] observed substantial improvements in the SRS-2 total score (F(2.12, 42.44) = 32.69, *p* < 0.001) and social awareness (F(2.34, 46.84) = 15.53, *p* < 0.001) when tDCS was combined with social cognition training, suggesting a moderate to high impact on social functioning.

#### Studies examining related social domains

In terms of other related social domains, various studies examined tDCS's impact on different aspects of social functioning. Nazari et al. [68] found significant improvements in emotion recognition (F = 50.23, *p* = 0.001, partial η^2^ = 0.71) following 10 sessions of tDCS, highlighting a large effect size and emphasizing its potential to enhance facial emotion recognition, a key component of social communication in children with ASD. Zemestani et al. [44] also reported significant improvements in communication (F1.4 = 79.51, *p* < 0.001, ηp^2^ = 0.51), ToM (F2.4 = 5.71, *p* = 0.007, ηp^2^ = 0.29), and emotion regulation (F1.8 = 5.02, *p* = 0.016, ηp^2^ = 0.24) with bilateral tDCS, indicating a moderate effect size in social-related domains. Han et al. [66] reported improvements in social functioning, particularly reductions in restricted and repetitive behaviors (RRBs), after 10 sessions of left DLPFC cathodal tDCS combined with cognitive remediation. While effect sizes and p-values were not specified, the findings suggested positive changes in social behaviors. In contrast, Wang et al. [72] observed improvements in social perception and interaction following HD-tDCS, though no detailed statistical data were provided. Other studies, such as Kang et al. [67] and Sun et al. [71], suggested neurophysiological changes, but their relevance to social functioning remained unclear due to the lack of direct social outcome measures.

### Analysis

#### Autism Treatment Evaluation Checklist (ATEC)

**Fig. 4:**
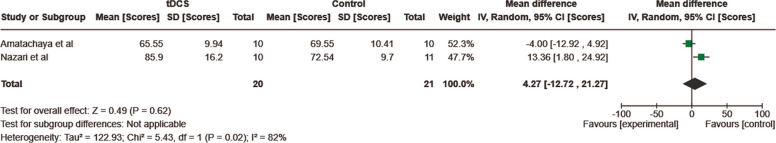
ATEC Scale active vs. sham/control tDCS comparison. A meta-analysis forest plot contrasting the effects on the ATEC scale of active against sham/control tDCS. Displayed are confidence ranges and pooled effect estimates

Data for the Autism Treatment Evaluation Checklist (ATEC) scale were pooled from two studies: Amatachaya et al. [63] and Nazari et al. [68]. The overall effect test yielded a Z-value of 0.49 (*P* = 0.62), indicating no significant difference between the tDCS and control groups. The studies reported considerable variation in mean differences: Amatachaya et al. [63] showed a mean difference of −4.00 (95% CI: −12.92, 4.92), while Nazari et al. [68] found a mean difference of 13.36 (95% CI: 1.80, 24.92), suggesting contrasting outcomes. The pooled mean difference was 4.27 (95% CI: −12.72, 21.27), emphasizing the lack of consistent effects of tDCS. High heterogeneity was observed, with Tau^2^ = 122.93, Chi^2^ = 5.43 (*P* = 0.02), and I^2^ = 82%, indicating substantial variability that could be attributed to differences in study designs or participant characteristics (Fig. 4).

#### ToM

**Fig. 5:**
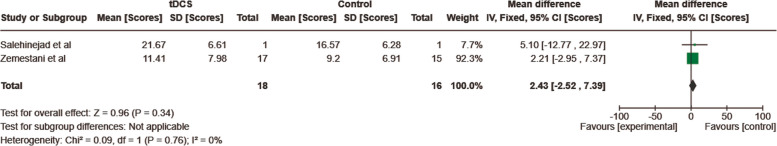
ToM Scale active vs. sham/control tDCS comparison. Visualizing the effect sizes across included studies, this forest plot contrasts the effects of active against sham/control tDCS on the ToM scale

For the ToM scale, data were pooled from Salehinejad et al. [13, 14] and Zemestani et al. [44]. The overall effect test yielded a Z-value of 0.96 (*P* = 0.34), suggesting no statistically significant difference between tDCS and control groups. The studies showed variability in mean differences: Salehinejad et al. [13, 14] reported a mean difference of 5.10 (95% CI: −12.77, 22.97), while Zemestani et al. [44] showed a mean difference of 2.21 (95% CI: −2.95, 7.37). The pooled mean difference was 2.43 (95% CI: −2.52, 7.39), suggesting a modest but inconsistent effect of tDCS on ToM. The heterogeneity was minimal, with Chi^2^ = 0.09 (*P* = 0.76) and I^2^ = 0%, indicating no significant variation between the studies (Fig. 5).

#### Social Responsiveness Scale (SRS)

**Fig. 6:**
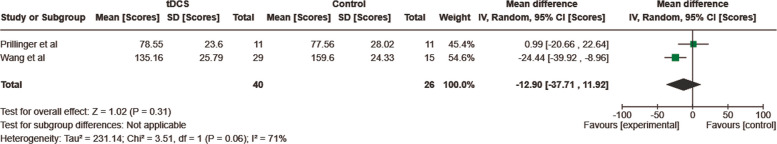
SRS Scale active vs. sham/control tDCS comparison. Summarizing research results and variability, the forest plot shows the effects of active against sham/control tDCS on the SRS

The pooled data for the Social Responsiveness Scale (SRS) were derived from Prillinger et al. [69] and Wang et al. [72]. The overall effect test showed a Z-value of 1.02 (*P* = 0.31), demonstrating no significant difference between tDCS and control groups. Individual study results showed Prillinger et al. [69] with a mean difference of 0.99 (95% CI: −20.66, 22.64), while Wang et al. [72] reported a mean difference of −24.44 (95% CI: −39.92, −8.96), favoring the control group. The pooled mean difference was −12.90 (95% CI: −37.71, 11.92), reinforcing the inconsistency in effects across studies. High heterogeneity was observed, with Tau^2^ = 231.14, Chi^2^ = 3.51 (*P* = 0.06), and I^2^ = 71%, suggesting significant variability in tDCS effects (Fig. 6).

### Publication bias

#### Autism Treatment Evaluation Checklist (ATEC)

**Fig. 7:**
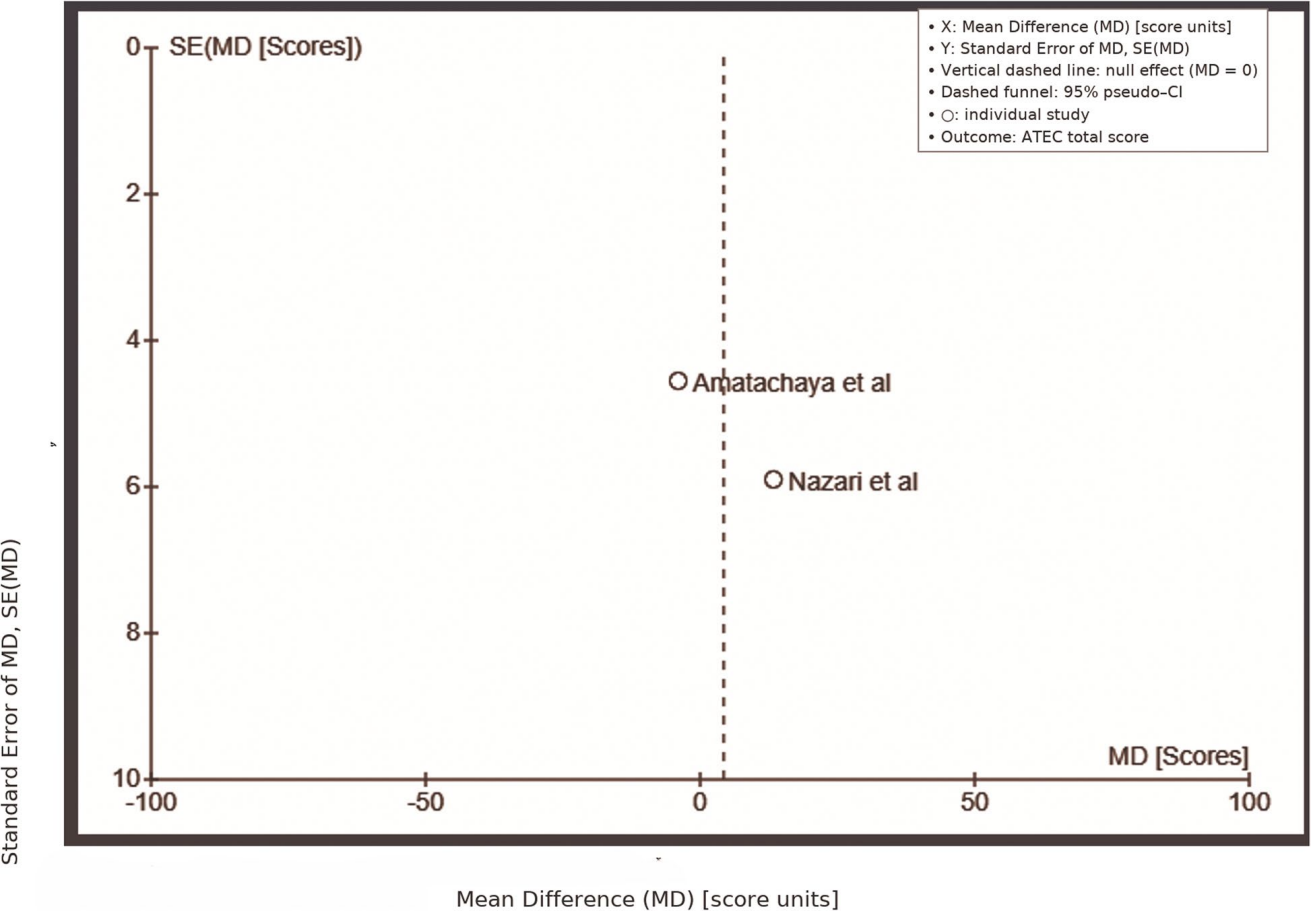
Funnel Plot: ATEC Scale. A funnel plot evaluating publication bias in studies looking at how tDCS affects the ATEC scale. Plot symmetry or asymmetry point to possible study selection bias

The funnel plot for the ATEC scale (Fig. 7) displayed a reasonably symmetric distribution, with studies by Amatachaya et al. [63] and Nazari et al. [68] distributed evenly around the mean difference axis. Though some dispersion was noted, there was no clear asymmetry. The mean differences ranged from −4.00 (Amatachaya et al.) to 13.36 (Nazari et al.), with standard errors between 2 and 8. This suggests no strong evidence of publication bias, and the results are unlikely to be influenced by selective publication.

#### ToM

**Fig. 8:**
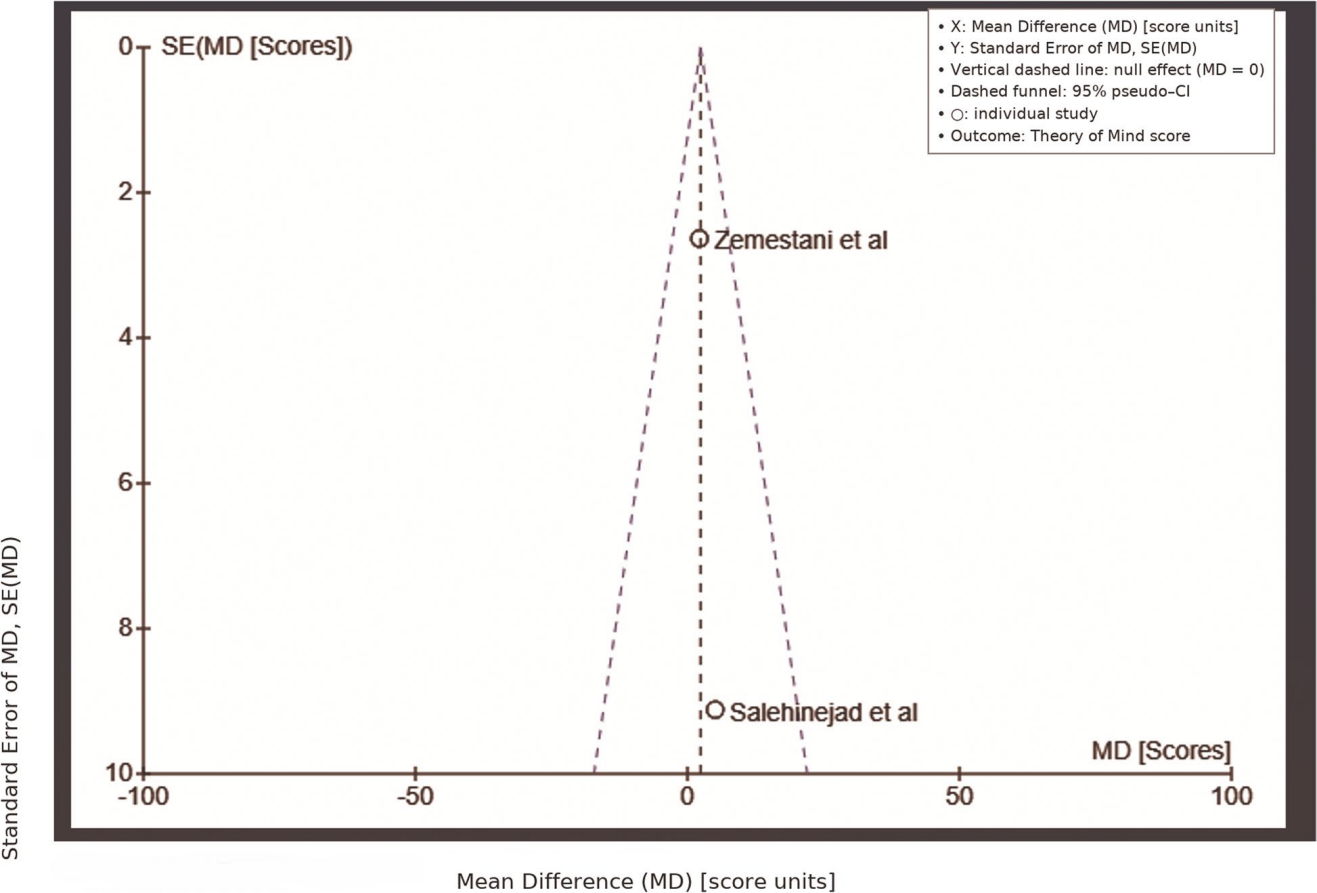
Funnel Plot: ToM Scale. A funnel diagram assessing publication bias for research on how tDCS influences the ToM scale

The funnel plot for ToM (Fig. 8) exhibited asymmetry, with studies by Salehinejad et al. [13, 14] and Zemestani et al. [44] placed on both sides of the mean difference axis. However, there was noticeable clustering on the right side, indicating a higher frequency of larger effect sizes. This suggests potential publication bias, where studies with smaller or non-significant results might be underrepresented in the literature.

#### Social Responsiveness Scale (SRS)

**Fig. 9:**
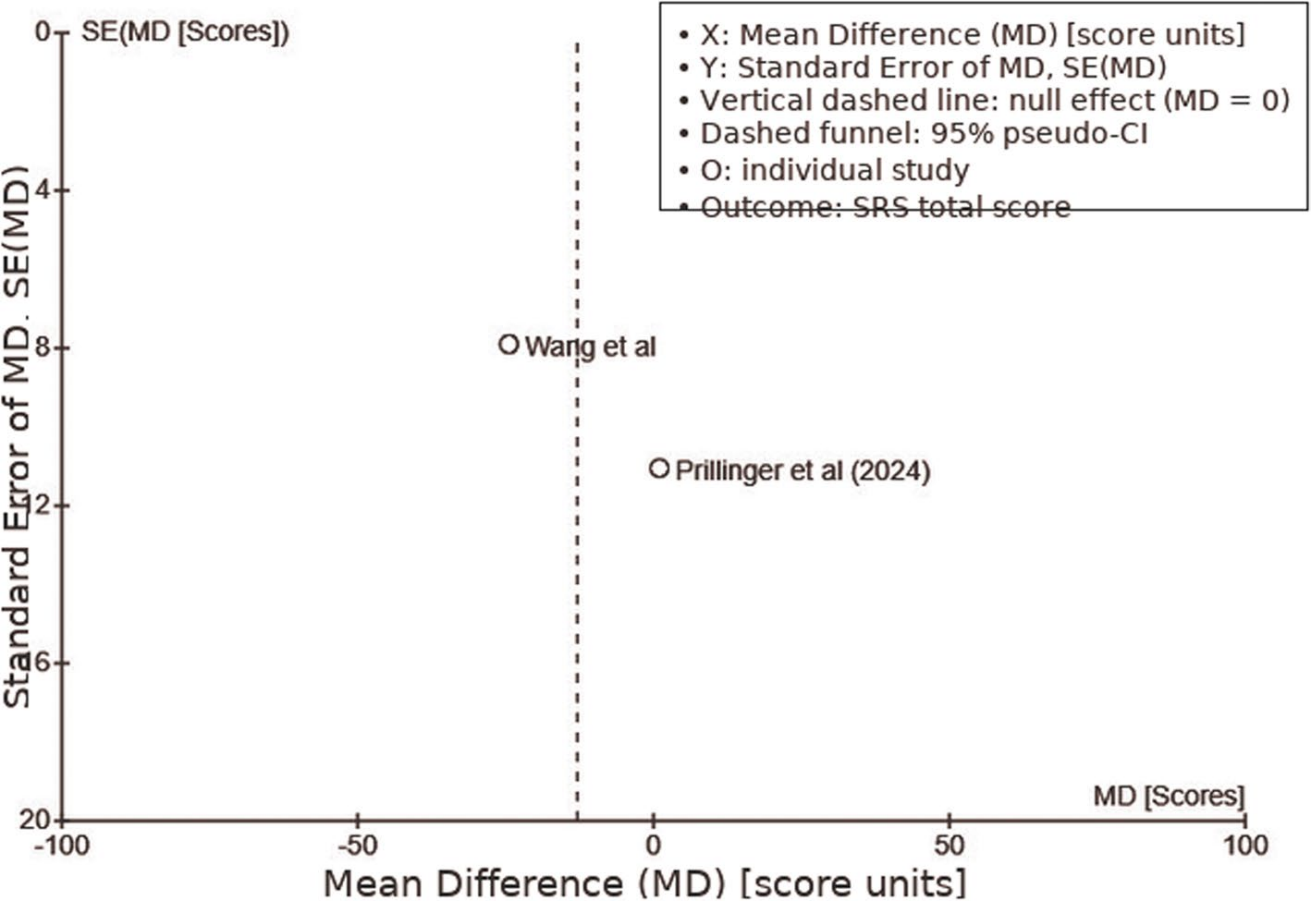
Funnel Plot: SRS Scale. A funnel plot assessing publication bias in studies investigating tDCS effects on the SRS

The funnel plot for the SRS (Fig. 9) revealed noticeable asymmetry, with fewer studies on the lower left side of the mean difference axis, where negative effects would appear. This indicates a possible publication bias, suggesting that studies with larger or positive effect sizes may be more likely to be published, while smaller or negative results may not be as widely disseminated.

### Certainty of evidence

The certainty of the evidence for social interactions, assessed using the ATEC, ToM, and SRS scales, is rated as "low" by the GRADE approach (Table 4). This rating is based on several factors: a moderate to high risk of bias in most studies, primarily due to challenges in blinding participants and personnel, as tDCS produces noticeable sensations that reveal treatment allocation; serious inconsistency between studies, such as varying participant age and gender, and differences in tDCS protocols (e.g., electrode montages, current intensity, duration, and session count); and serious imprecision due to a lack of consistent effects across studies, with all plausible residual confounding suggesting a spurious effect. Despite these concerns, no effect was observed in the pooled analysis. The number of studies included was six randomized trials, with a total of 86 participants in the tDCS group and 70 in the sham group. The effect size was reported as a mean 100 score higher (95% CI: 37.71 lower to 21.27 higher), with a relative effect of ⨁◯◯◯ and classified as critical in importance.

**Table 4:** GRADE Approach: Evidence Profile for Meta-analysis

Certainty assessment							№ of patients		Effect		Certainty	Importance
**№ of studies**	**Study design**	**Risk of bias**	**Inconsistency**	**Indirectness**	**Imprecision**	**Other considerations**	**tDCS**	**Sham**	**Relative** **(95% CI)**	**Absolute** **(95% CI)**		
**Social interactions (follow-up: mean 1 days; assessed with: ATEC, ToM, SRS; Scale from: 1 to 160)**												
6	randomised trials	serious^a^	serious^b^	not serious	serious^c^	all plausible residual confounding would suggest spurious effect, while no effect was observed	86	70	-	mean **100 score higher** (37.71 lower to 21.27 higher)	⨁◯◯◯ low^a,b,c^	CRITICAL

*CI* confidence interval

## Discussion

This systematic review and meta-analysis aimed to explore the effects of transcranial direct current stimulation (tDCS) on social cognition in children with autism spectrum disorder (ASD). The findings suggest that tDCS, particularly when applied to the DLPFC, holds promise in improving various aspects of social functioning, including emotion recognition, ToM, and social responsiveness. However, the current findings show low-grade evidence due to methodological and experimental factors. Across the included trials, follow-up assessments were concentrated immediately post-stimulation and within the first 1–4 weeks. Immediate effects were consistently observed and were generally maintained into the short-term (≤ 1 week), particularly after multi-session courses (e.g., 5–20 sessions over 1–4 weeks). Evidence for durability at 2–4 weeks was more variable and imprecise due to fewer contributing studies, and follow-up beyond 4 weeks was rare, limiting conclusions about longer-term maintenance.

The predominance of evidence for increased cortical excitability with anodal tDCS comes from studies targeting the primary motor cortex (M1) because M1 offers robust, quantifiable neurophysiological endpoints—most notably, motor evoked potentials (MEPs) elicited by transcranial magnetic stimulation (TMS) [73, 74]. These MEPs provide a direct, sensitive, and reproducible measure of changes in cortical excitability, facilitating mechanistic studies and protocol optimization [74]. The motor system’s well-characterized anatomy and functional output further enable precise mapping of stimulation effects, which is less straightforward in non-motor regions [73, 75].

Generalizing these findings to other brain regions, such as the DLPFC, is not straightforward. The neurophysiological and behavioral effects of tDCS are region-specific, influenced by differences in cytoarchitecture, baseline activity, and network connectivity [26]. Direct comparisons using multimodal TMS-EEG and neuroimaging approaches demonstrate that the effects observed in M1 do not transfer one-to-one to the DLPFC. For example, cathodal tDCS over M1 and DLPFC produces distinct patterns of TMS-evoked potentials and oscillatory changes, with DLPFC showing more uniform reductions in early TEP peaks across dosages, and less pronounced effects on late TEPs and oscillations [75]. Similarly, studies using anodal tDCS over DLPFC report modulation of functional connectivity and cerebral perfusion, but the magnitude and spatial distribution of these effects differ from those seen in M1, and behavioral outcomes are often less robust or more variable [76–78].

Meta-analytic evidence further supports the notion that stimulation effects in DLPFC are task- and protocol-dependent, with significant improvements in executive function observed only under specific conditions, such as when using extracranial cathodes or smaller anodes [79]. Multimodal studies combining behavioral, neurochemical, and imaging endpoints have found that tDCS effects in DLPFC and temporo-parietal regions are often subtle and may not mirror the clear excitability changes seen in M1, emphasizing the need for region-specific mechanistic investigations [80]. In summary, while the foundational evidence for tDCS-induced increases in cortical excitability is strongest for M1 due to methodological advantages, extrapolation to regions like the DLPFC requires caution [81]. The effects of tDCS are highly dependent on the targeted region’s neurophysiology, the stimulation protocol, and the outcome measures employed, as demonstrated by direct comparative and meta-analytic studies [75–77, 79, 80].

Most of the reviewed studies have applied anodal tDCS over the left DLPFC and consistently yield significant enhancements in emotion recognition and broader social-cognition outcomes [44, 67, 71]. For instance, Zemestani et al. [44] demonstrated positive effects on communication, ToM, and emotion regulation when stimulating the left DLPFC, further supporting the critical role of this region in social cognitive functions. However, not all studies found equally robust effects, particularly when using cathodal or mixed stimulation protocols. Interestingly, other studies employed cathodal or combination montages, such as stimulating both the left and right DLPFC (dual stimulation) or targeting regions like the right temporoparietal junction (rTPJ) and medial prefrontal cortex (mPFC). These variations in stimulation protocols have led to mixed results. For example, research by Han et al. [65] using cathodal stimulation over the right supraorbital area showed significant improvements in social responsiveness, though the effects were less pronounced compared to those observed with anodal stimulation. The combination of anodal and cathodal stimulation (dual stimulation) was observed to have boosting effects in some cases. In the study by Zemestani et al. [44], a mixed stimulation protocol involving both the left and right DLPFC showed positive effects across multiple domains, including communication, ToM, and emotion regulation strategies. This suggests that dual stimulation may offer a more comprehensive approach to modulating brain activity and improving social cognition.

It is also important to note that different stimulation parameters (e.g., intensity, session duration, frequency) varied across studies, contributing to the inconsistency in results. While most studies used a stimulation intensity of 1–1.5 mA for durations ranging from 15 to 20 min, the frequency and total number of sessions varied greatly. For instance, Kang et al. [67] administered 10 sessions, spaced every other day, while Prillinger et al. [70] used 10 sessions spread over two weeks. Such variations make it difficult to identify an optimal tDCS protocol for social cognitive improvements, though longer, more frequent sessions may be beneficial for producing more significant effects. Additionally, some studies explored the impact of high-definition tDCS (HD-tDCS) and found that these more targeted approaches could provide superior outcomes in improving social cognition. For example, Wang et al. [72] utilized HD-tDCS with central anodes placed at Cz and F3, demonstrating improvements in the Social Responsiveness Scale (SRS) domains and autism-related behaviors. While these results suggest that HD-tDCS may offer additional benefits, further research is needed to directly compare its efficacy to conventional tDCS approaches.

tDCS modulates cortical excitability through mechanisms involving both metabotropic glutamate receptors (mGluRs) and N-methyl-D-aspartate (NMDA) receptors. Cathodal tDCS has been shown to induce long-term depression (LTD) of excitatory synaptic strength in neocortical tissue, a process that is critically dependent on mGluR5 signaling and downstream mechanistic target of rapamycin (mTOR)-mediated protein synthesis but notably persists even when NMDA receptors are inhibited [82]. This suggests that mGluR5-dependent pathways are central to certain forms of tDCS-induced plasticity, and pharmacological facilitation of mGluR5 can enhance the durability of these effects [82]. In contrast, anodal tDCS-induced long-term potentiation (LTP)-like plasticity is more closely associated with NMDA receptor activity, and can be modulated by dopaminergic signaling, particularly via D2 receptor-dependent reduction of NMDA receptor function. ASD genetics frequently converge on synaptic dysfunction, with aberrant NMDA receptor-mediated plasticity and altered mGluR signaling implicated in both syndromic and non-syndromic forms of ASD. Mutations in genes regulating synaptic proteins, glutamatergic transmission, and the E/I balance can result in abnormal cortical circuit development and function, leading to atypical responses to interventions that target these pathways. Therefore, it is critical to mention that the effects of tDCS on cortical tissue in individuals with ASD may differ from those observed in neurotypical controls or other neurodevelopmental syndromes, due to underlying disruptions in NMDA receptor-dependent plasticity and mGluR5 signaling [82, 83]. Altogether, this highlights the need for mechanistic studies and individualized approaches when considering tDCS as a therapeutic modality in genetically defined ASD populations.

Some limitations should be considered when drawing conclusions. The studies had methodological issues, including small sample sizes (10 to 60 participants) and less robust designs like pre-post or single-case studies, which contribute to variability in the results and affect the generalizability of the findings.

## Conclusion

tDCS shows promise as an adjunctive intervention for enhancing social cognition in children with ASD, particularly when targeting the left DLPFC with anodal stimulation. Studies have demonstrated significant improvements in emotion recognition, ToM, and social responsiveness with this approach. To optimize its effectiveness, future studies should focus on large-scale randomized controlled trials with standardized protocols and better blinding techniques. Identifying the most effective stimulation parameters and combining tDCS with other therapeutic approaches, such as cognitive and social skills training, is crucial for enhancing its clinical application.

## Data Availability

Data is available upon reasonable request.

## Glossary

Autism Spectrum Disorder (ASD): This is the primary focus of systematic review and meta-analysis.
Transcranial Direct Current Stimulation (tDCS): This is the intervention being investigated for its potential to improve social interaction in children with ASD.
Dorsolateral Prefrontal Cortex (DLPFC): This is the most common target for tDCS stimulation in the included studies, as it is thought to play a role in social cognition and executive functions.
Temporoparietal Junction (TPJ): Another brain region involved in social cognition that may be targeted by tDCS.
Social Responsiveness Scale (SRS-2): A commonly used outcome measure to assess social functioning in individuals with ASD.
Autism Treatment Evaluation Checklist (ATEC): Another outcome measure used to assess various aspects of ASD, including social interaction.
Theory of mind (ToM): A cognitive ability that allows individuals to understand and attribute mental states to others, an essential skill for social interaction, particularly in individuals with ASD.
